# Socio-demographic predictors and average annual rates of caesarean section in Bangladesh between 2004 and 2014

**DOI:** 10.1371/journal.pone.0177579

**Published:** 2017-05-11

**Authors:** Md. Nuruzzaman Khan, M. Mofizul Islam, Asma Ahmad Shariff, Md. Mahmudul Alam, Md. Mostafizur Rahman

**Affiliations:** 1 Department of Population Science, Jatiya Kabi Kazi Nazrul Islam University, Mymensingh, Bangladesh; 2 Department of Public Health, La Trobe University, Melbourne, Australia; 3 Centre of Foundation Studies in Science, University of Malaya, Kuala Lumpur, Malaysia; 4 Department of Statistics, University of Rajshahi, Rajshahi, Bangladesh; 5 Department of Population Science and Human Resource Development, University of Rajshahi, Rajshahi, Bangladesh; BRAC, BANGLADESH

## Abstract

**Background:**

Globally the rates of caesarean section (CS) have steadily increased in recent decades. This rise is not fully accounted for by increases in clinical factors which indicate the need for CS. We investigated the socio-demographic predictors of CS and the average annual rates of CS in Bangladesh between 2004 and 2014.

**Methods:**

Data were derived from four waves of nationally representative Bangladesh Demographic and Health Survey (BDHS) conducted between 2004 and 2014. Rate of change analysis was used to calculate the average annual rate of increase in CS from 2004 to 2014, by socio-demographic categories. Multi-level logistic regression was used to identify the socio-demographic predictors of CS in a cross-sectional analysis of the 2014 BDHS data.

**Result:**

CS rates increased from 3.5% in 2004 to 23% in 2014. The average annual rate of increase in CS was higher among women of advanced maternal age (≥35 years), urban areas, and relatively high socio-economic status; with higher education, and who regularly accessed antenatal services. The multi-level logistic regression model indicated that lower (≤19) and advanced maternal age (≥35), urban location, relatively high socio-economic status, higher education, birth of few children (≤2), antenatal healthcare visits, overweight or obese were the key factors associated with increased utilization of CS. Underweight was a protective factor for CS.

**Conclusion:**

The use of CS has increased considerably in Bangladesh over the survey years. This rising trend and the risk of having CS vary significantly across regions and socio-economic status. Very high use of CS among women of relatively high socio-economic status and substantial urban-rural difference call for public awareness and practice guideline enforcement aimed at optimizing the use of CS.

## Introduction

Caesarean Section (CS) is a surgical procedure to prevent poor obstetric outcomes and can be life-saving for both mother and fetus [1]. CS reportedly prevents approximately 187,000 maternal and 2.9 million neonatal deaths annually worldwide [1, 2]. However, unnecessary CS presents risks for both women and neonates [3, 4]. On the basis of findings from the survey of 373 facilities across 24 countries in 2008, the World Health Organization (WHO) concluded that unnecessary CS increases the risk of maternal mortality and morbidity, neonatal death, neonatal admission to intensive care [5]. These findings were recently supported by a hospital based prospective study, which used data from nine countries in the wider Asian region (Bangladesh, China, Indonesia, Mongolia, Myanmar, Nepal, Thailand, Sri Lanka, Vietnam) [6]. In addition to potential adverse health consequences, unnecessary CS also causes a substantial economic burden on individual, family and overall society [7]. The estimated cost of post-partum medical care and re-hospitalization associated with unnecessary CS is approximately US$ 2.32 billion globally [7].

Over the past few decades there has been upward trend in global CS rates [8]. While very low and very high rates of CS can be harmful, an optimum rate is unknown. According to WHO, 5–15% is a reasonable range estimate [4] until further research produce a better estimate. In 2014 around 18% of the world’s births were delivered by CS [8]. The highest rate of CS (32%) was reported in Latin America and the Caribbean region, while the African region reported the lowest rate (7%) [8]. A recent analysis of combined data of demographic and health surveys of the 43 Asian and African countries found higher rate of CS among the urban rich and a lower rate among the rural poor women [9].

Several studies, mainly from high- and middle-income countries, examined the determinants of CS use, but results were conflicting [10–12]. A recent cohort study in the United States showed that prior CS was the strongest indication of CS operation [13]. A systematic review of 17 studies found maternal choice was the strongest indication of CS [14]. Two regional studies in Bangladesh found maternal education, age, birth order and prolonged labour were significant factors for caesarean delivery [11, 15]. However, these studies did not explore the variation in CS across different socio-demographic variables and were limited to only some specific determinants. Moreover, instead of national data only regional data were used. It is important that regional variation is accounted for, otherwise the national rate can mask substantial degree of intra socio-geographic variation. Identification of factors, while adjusted for regional variation, can inform appropriate measures in rationalizing utilization of CS.

Bangladesh has achieved remarkable success in improving maternal and child health. The majority (79%) of the Bangladeshi women now receive antenatal care, and 36% receive post-natal care [16]. In 2014, overall 37% of births were delivered informal healthcare facilities including 22% births in the private facilities, of which a staggering 61% and 77% of births ended in CS, respectively [16, 17]. A number of factors may influence this increasing rate of CS in Bangladesh, including high rate of adolescent pregnancy (35%), increasing rate of late aged pregnancy (5%), improving educational and socio-economic status of mothers, and the ongoing dual nutritional burden (co-existing conditions of under and over nutrition) [15, 18]. However, there is no clear indication as to which socio-demographic groups are experiencing the relative upward or downward trends in utilization of CS, and if these trends are influenced by factors such as locations. It is important, therefore, to carry out a comprehensive analysis of the relative rate of change in the prevalence of CS in Bangladesh and identify the factors influencing this change. The primary objective of this study was to (i) determine change over time in the average annual rates of CS by selective socio-demographic factors, and (ii) identify the significant socio-demographic factors of CS in 2014.

## Methods

### Study design and data sources

We analyzed four waves of data of Bangladesh Demographic and Health Surveys (BDHSs), which were conducted between 2004 and 2014. These nationally representative cross-sectional household surveys were conducted approximately every three years in all seven administrative regions (divisions): Barisal, Chittagong, Dhaka, Khulna, Rajshahi, Rangpur and Sylhet–covering both rural and urban areas. Each division is sub-divided into districts and each district into sub-districts (Upazilas), which are further divided into rural and urban areas. Each wave of these surveys used two-stage cluster sampling whereby enumeration areas (clusters) were first drawn from the national population and housing census sampling frame conducted in 2001 and 2011 by Bangladesh Bureau of Statistics [16, 19–21]. In the first stage of sampling, 600 primary sampling units were selected, with the probability of selection proportional to the unit size. In the second stage, 30 households were selected within each primary sampling unit by systematic random sampling. Further details about sampling design and other related issues of BDHSs can be found elsewhere [16, 19–21]. The overall response rate was around 98%. These surveys are periodically conducted by the National Institute of Population Research and Training where the United States Agency for International Development provided financial support and ICF International of Calverton, USA offered technical support. National Research Ethics Committee in Bangladesh reviewed and approved the survey protocol. Data collection procedures were also approved by the ORC Macro (Macro International Inc) Institutional Review Board. Informed consent was obtained from all participants. The BDHSs collect a range of information including intimate partner violence. Because the existence of a signed consent form can provide a risk in itself for the abused person, oral informed consent was obtained from respondents by interviewers. The ethics committee approved this consent procedure.

These surveys were conducted to collect data on a wide range of population for monitoring a range of indicators including health and nutrition. Detailed information on the socio-demographic characteristics of all participants were collected by trained staff. Each wave of these surveys used standard questionnaire and little or no differences exist between the questionnaires across the waves.

### Outcome variable

Our primary outcome was the method of pregnancy delivery. The BDHS collected a range of birth information including baby weight, method of pregnancy delivery, and type of pregnancy complication from each of the respondents who were of at least 15 years old and who gave birth within the last three years prior to the survey. Respondents were also asked about the method of pregnancy delivery for their last child and were flagged if caesarean delivery was reported.

### Explanatory or independent variables

A range of socio-demographic variables were used in this study based on previous research demonstrating the importance of these factors [11, 22–24]. The potential determinants were the age of women during delivery (≤19, 20–34, ≥35 years), respondent’s place of residence (rural, urban), wealth quintile (poorest, poorer, middle, richer, richest) [25], region (Barisal, Chittagong, Dhaka, Khulna, Rajshahi, Rangpur, Sylhet), interval between deliveries (≤2 year, 3–4 year, ≥5 year), number of births delivered (≤2, 3–4, ≥5), maternal body mass index (underweight, normal weight, overweight, obese) and the number of antenatal visits (no visits, 1–4 visits, >4 visits). Level of education was described in mean years of schooling completed, and was used as a categorical variable (no formal education, primary, secondary, higher) in regression models.

### Statistical analysis

Rate of change analysis was performed by using four successive BDHS data conducted in 2004, 2007, 2011 and 2014. For this analysis the average annual rate of increase (AARI) was calculated by using the formula *Y*_*t*+*n*_ = *Y*_t_* (1 + *r*)^*n*^ for caesarean delivery in which *Y*_*t*_ = prevalence of caesarean delivery of any given year, r = annual rate of change, n = number of years between two surveys, and *Y*_*t*+*n*_ = prevalence of caesarean delivery of the (*t* + *n*)^*th*^ year. This formula was adopted and modified based on the information provided in the UNICEF technical note [26]. In this dataset individual women were nested within household, households were nested within cluster/primary sampling unit, and clusters were nested within regions. To account for this multiple hierarchy and dependency in data, we performed multi-level logistic regression to assess the factors associated with CS recorded in 2014 dataset. Additionally, we performed likelihood test to choose preferable models. The tests compared random effects model against fixed effects model and found statistically significant results (*p*<0.05). This implies the random effect models are necessary for modeling this data. Both unadjusted and adjusted models were presented for each of the socio-economic determinants. The initial model included only specific determinants of CS, and the final model was adjusted for all potential confounding factors. All analyses accounted for probability sample design. Stata software (version13.1) was used for all statistical analyses.

## Results

### Sample characteristics

After excluding the non-qualified respondents and missing data, 4,726 individuals remained in the sample for analysis in 2014 dataset. The crude and age standardized characteristics of the study sample are shown in Table 1. The average age of participants was 25 years (± 5.7), weight was 55 kg (± 9.6) and age at marriage was around 16 years (± 2.9). Mean number of antenatal visits was around three (±3.8) during pregnancy.

**Table 1:** Study population characteristics (based on 2014 dataset).

Characteristics	Subjects (N)	Crude	Age -standardized
**Mean (SD)**			
Maternal age, years	4726	24.6 (5.7)	24.92 (4.9)
Weight (in Kg)	4724	55.36 (9.6)	55.32 (7.5)
Education, years	4726	6.27 (3.8)	6.63 (3.6)
Children ever born	4726	2.20 (1.4)	2.67 (1.3)
Age at first marriage	4726	16.35 (2.9)	16.43 (2.4)
Number of antennal visit	4485	2.77 (3.8)	2.94 (2.7)

### Change in CS over time

**Fig 1:**
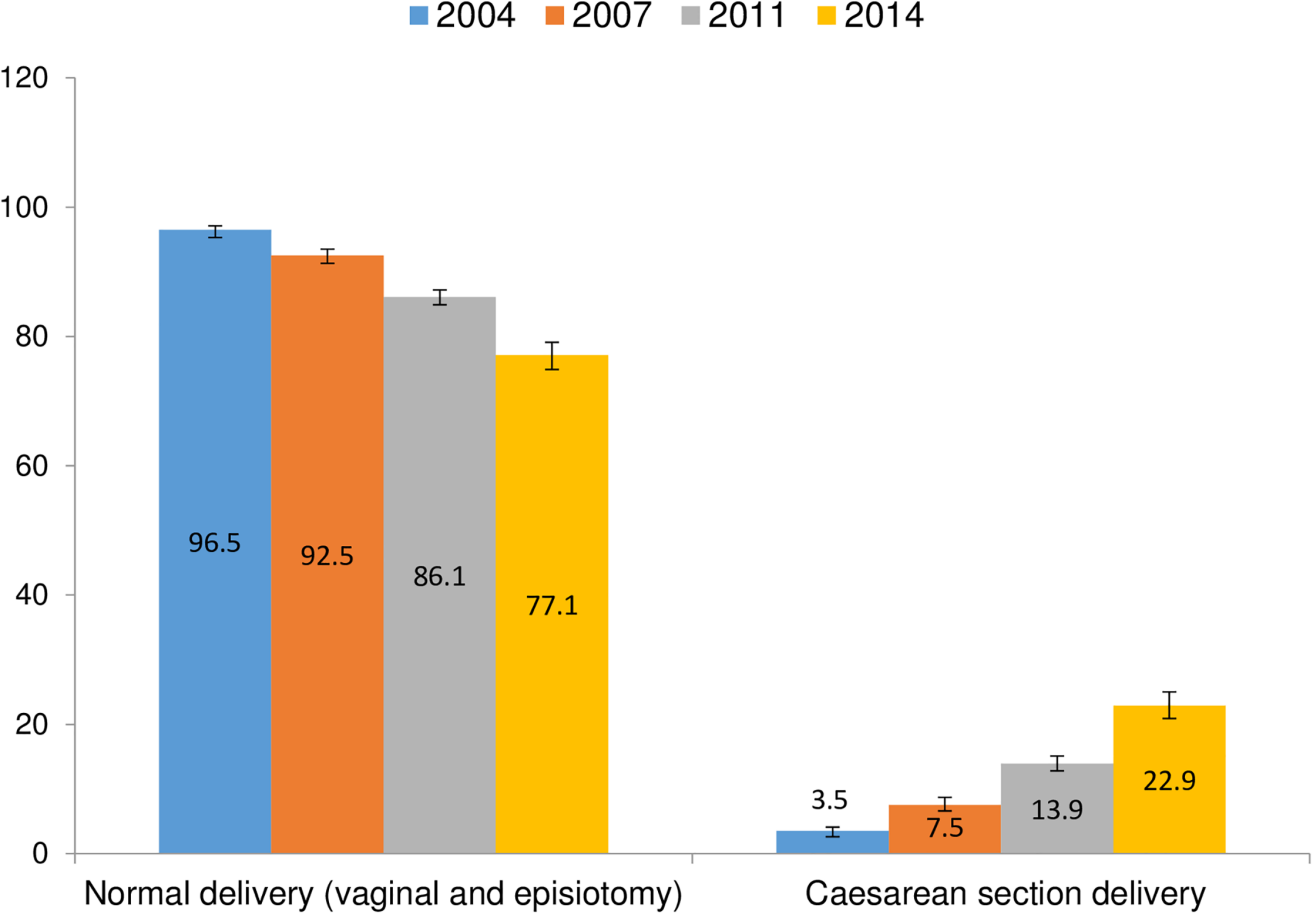
Trend of caesarean delivery over the survey years.

Table 2 shows the AARI in the prevalence of CS in Bangladesh. The rate of CS has increased by around 19% from 2004 to 2014 (Fig 1). There was a higher change in the annual rate of CS among women aged ≤19 years (6.23%) and ≥35 years (7.71%) compared to the women aged 20–34 years (3.77%). Urban women reported a higher AARI of CS than rural women (11.81% *vs* 3.55%). Women in the Khulna region reported a higher AARI in CS (16.83%) than the women in other regions. In addition, an overall increase in the prevalence of CS was observed across different wealth quintiles between 2004 and 2014. The observed increase was highest among the richest (33.06%) and lowest among women in the poorest households (0.74%). Average annual rate of CS apparently increased in tandem with the level of education. Between 2004 and 2014 the total AARI of CS was highest (13.70%) among higher educated women and lowest (0.86%) among women who did not have any formal education. The AARI in the prevalence of CS was higher among the women who gave birth to two or less babies (6.70%) than women who gave birth more than two children. The prevalence of CS increased across the BMI categories. A higher AARI was observed among women who were obese (25.42%).

**Table 2:** Average annual rate of increase (AARI) in the prevalence of caesarean delivery in Bangladesh, 2004–2014.

	2004	2007	Percent AARI (2004–2007)	2011	Percent AARI (2007–2011)	2014	Percent AARI (2011–2014)	Percent AARI (total, 2004–2014)
**Maternal age (in years)**								
≤19	3.80	6.79	1.11	11.44	2.20	23.58	19.80	6.23
20–34	4.60	8.89	1.92	15.65	4.42	20.23	2.14	3.77
≥35	3.45	7.63	1.84	11.92	1.92	25.09	25.91	7.71
**Type of residence**								
Rural	1.76	4.76	1.12	10.60	3.31	16.92	3.85	3.55
Urban	10.43	15.55	2.60	24.20	7.69	35.95	17.87	11.83
**Region**								
Barisal	3.19	6.07	1.05	12.53	4.03	19.24	4.35	3.98
Chittagong	2.86	7.31	2.04	12.11	2.32	19.17	4.84	4.11
Dhaka	6.48	11.64	2.63	17.18	2.99	31.62	35.97	11.35
Khulna	5.31	11.92	4.22	23.20	15.78	34.12	14.33	16.83
Rajshahi	3.44	8.90	2.92	16.18	5.17	26.48	12.13	9.01
Rangpur^¶^				10.89	14.22	19.33	7.25	5.91^╬^
Sylhet	4.67	5.19	0.14	13.07	6.17	12.38	-0.16	1.16
**Wealth classification**								
Richest	15.85	26.23	12.40	38.76	21.93	51.13	21.03	33.06
Richer	3.10	8.22	2.60	16.78	7.50	28.85	19.44	12.13
Middle	1.62	2.78	0.34	10.55	5.98	18.83	6.92	4.59
Poorer	0.91	1.48	0.15	6.34	2.37	11.21	2.38	1.80
Poorest	0.06	1.80	0.54	2.92	0.32	5.54	0.93	0.73
**Education**								
No formal education	0.75	0.84	0.02	3.08	0.75	6.97	1.64	0.86
Primary(completed grade 5)	2.05	2.76	0.19	7.29	2.10	11.63	1.96	1.61
Secondary(completed grade 10)	6.80	12.90	3.60	18.10	2.67	26.30	6.77	6.03
Higher(completed higher secondary or further)	29.23	40.63	16.29	55.59	41.10	56.11	0.14	13.70
**Husband education**								
No formal education	0.83	1.43	0.16	4.12	0.96	8.46	1.96	1.14
Primary (completed grade 5)	1.86	3.49	0.50	8.65	2.63	14.37	3.18	2.49
Secondary (completed grade 10)	5.87	11.99	3.62	17.92	3.40	27.97	11.34	8.12
Higher (completed higher secondary or further)	20.91	32.97	19.39	43.89	14.33	51.95	6.50	21.29
**Children ever born**								
≤2	7.20	13.02	3.28	19.60	4.18	27.61	6.41	6.70
3–4	2.21	4.14	0.62	9.36	2.69	14.17	2.33	2.31
>4	0.76	1.13	0.10	2.11	0.28	6.91	2.32	0.85
**Maternal body mass index**								
Underweight (<18.5 kg/m^2^)	1.64	3.00	0.40	7.14	1.82	12.60	2.92	1.99
Normal weight (18.5–24.9 kg/m^2^)	4.01	7.86	1.62	13.35	2.95	21.37	6.43	4.67
Overweight (25.0–29.9 kg/m^2^)	21.45	30.06	7.61	35.86	3.26	41.39	2.98	6.34
Obese (≥30 kg/m^2^)	16.34	39.73	345.37	54.36	37.76	49.08	-0.73	25.42
**Total**	**3.50**	**7.50**	**1.72**	**13.90**	**3.95**	**22.98**	**8.68**	**6.01**

Data from BDHS 2004 [21]; BDHS 2007 [20], BDHS 2011 [19] and BDHS 2014 [16]

^¶^Rangpur division data were available for 2011 and 2014 survey waves

^╬^AARI from 2004–2014

### Socio-economic determinants of CS

**Fig 2:**
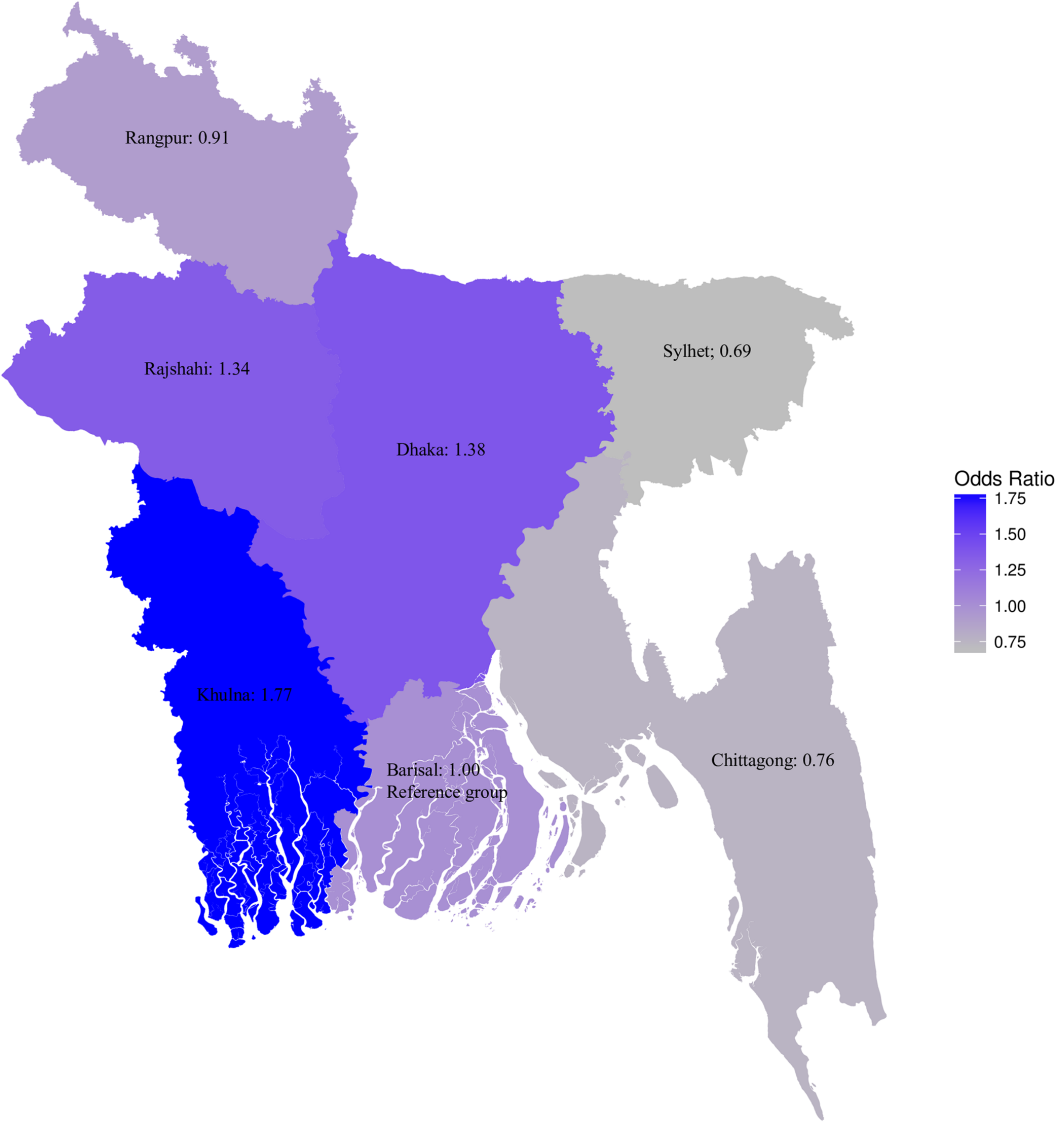
Likelihood of caesarean delivery by region.

The results of the multi-level logistic regression analysis assessing the determinants of CS are presented in Table 3. Participants aged ≥35 years were more likely to use CS (aOR, 1.98; 95% CI, 1.20–3.24) than the women aged 20–34 years. This risk was 1.22 times (95% CI, 1.00–1.48) higher among younger mothers (age ≤19 years) than those were 20–34 years old. Urban women reported a higher probability of having a CS (aOR, 1.24; 95% CI, 1.00–1.53) than rural women. Women in Khulna (aOR, 1.77; 95% CI, 1.22–2.56) region reported a higher use of CS than Barisal (Fig 2). Women from poorer and poorest households reported a lower use of CS at around 30% (aOR, 0.70; 95% CI, 0.51–0.95) and 54% (aOR, 0.46; 95% CI, 0.32–0.65), respectively, than women from middle income. The use of CS was 1.32 times (95% CI, 1.04–1.68) and 2.33 times (95% CI, 1.78–3.05) higher among the richer and richest households, respectively, than those within the middle wealth quintile.

**Table 3:** Odds ratio of using caesarean section in multilevel logistic regression analysis.

Variables	Adjusted odds ratio (95% CI)	*p*-value
**Maternal age, years**		
≤19	1.22 (1.00–1.48)	0.04
20–34	1.00	
≥35	1.98 (1.20–3.24)	0.01
**Type of residence**		
Rural	1.00	
Urban	1.24 (1.00–1.51)	0.05
**Region**		
Barisal	1.00	
Chittagong	0.76 (0.51–1.12)	0.15
Dhaka	1.38 (0.95–2.00)	0.08
Khulna	1.77 (1.22–2.56)	<0.01
Rajshahi	1.34 (0.92–1.96)	0.13
Rangpur	0.91 (0.60–1.39)	0.67
Sylhet	0.69 (0.47–1.03)	0.67
**Wealth classification**		
Richest	2.33 (1.78–3.05)	<0.01
Richer	1.32 (1.04–1.68)	0.02
Middle	1.00	
Poorer	0.70 (0.51–0.95)	0.02
Poorest	0.46 (0.32–0.65)	<0.01
**Education**		
No formal education	1.00	
Primary (completed grade 5)	1.28 (0.87–1.88)	0.20
Secondary (completed grade 10)	1.96 (1.35–2.87)	<0.01
Higher (completed higher secondary or further)	3.86 (2.51–5.93)	<0.01
**Birth interval**	
≤2	0.83 (0.54–1.27)	0.38
3–4	1.00	
>4	1.28 (1.00–1.63)	0.05
**Children ever born, number**		
≤2	1.50 (1.18–1.93)	<0.01
3–4	1.00	
>4	0.82 (0.48–1.40)	0.47
**Body weight**	
Underweight (<18.5 kg/m^2^)	0.76 (0.60–0.97)	0.03
Normal weight (18.5–24.9 kg/m^2^)	1.00	
Overweight (25.0–29.9 kg/m^2^)	1.66 (1.34–2.06)	<0.01
Obese (≥30 kg/m^2^)	1.95 (1.34–2.84)	<0.01
**Number of antenatal visit**		
No visit	1.00	
1–4	4.09 (2.81–5.93)	<0.01
>4	6.14 (4.08–9.24)	
Variance (cov.) of random effect		<0.01
Level 2 (household)	3.192e^-15^ (5.25e^-12^)	
Level 3 (community)	0.25 (0.07)	
LR test	16.25 (0.0003)	

Compared to women with no formal education, the likelihood of undergoing CS is significantly high for women with higher education (aOR, 3.86; 95% CI, 2.51–5.93) and secondary education (aOR, 1.96; 95% CI, 1.35–2.87). Participants with two or less children were more likely (aOR, 1.50; 95% CI, 1.18–1.93) to undergo CS than those with 3–4 children. The rate of CS increased in tandem with the number of antenatal visits. Women who made more than four antenatal visits were 6.14 times (95% CI, 4.08–9.24) more likely and those who made 1–4 visits were 4.09 times (95% CI, 2.81–5.93) more likely to report using CS in their recent delivery than the participants who did not make any antenatal visits. Maternal overweight and obesity were associated with undergoing CS by around 1.66 times (95% CI, 1.41–2.83) and 1.95 times (95% CI, 1.34–2.84), respectively, in comparison to women with normal weight. Underweight was found to be protective (aOR, 0.76; 95% CI, 0.60–0.97) of CS.

## Discussion

Using national data in Bangladesh this study assessed the recent changes of CS rate, and examined a range of socio-demographic characteristics that are likely to be determinants of CS use. One of the major strengths of this study is its well-designed nationwide survey and a high response rate. Overall, around 23% of babies were delivered via CS. Rate of CS has increased by around 19% between 2004 and 2014. This study also found younger (≤19) and advanced maternal age (≥35), urban place of residence, high socio-economic status, higher education, few children delivered (≤2), more antenatal visits, and maternal overweight and obesity were the key factors of the increasing rate of CS use.

The results presented here suggest a noticeable increase in CS rate over the survey years. In 1994 WHO recommended upper limits of CS rates at 15% and cautioned against rates between 1% and 5% in order to avoid death and severe morbidity [27–29]. This recommendation is consistent to findings of some previous studies that found 5–10% of CS had improvement in terms of maternal and neonatal outcome in population level [30–32] and no significant effect thereafter [33]. Clearly, the most recent rate of CS, as observed in 2014 survey, is much higher than the WHO recommended upper limit. Also, if this trend continues then it is very likely that CS will bring more harms than benefits at the population level.

Our finding regarding higher rate of CS among urban women is similar to the study findings in other developing countries. For example, in a retrospective analysis of data from demographic and health survey conducted in Pakistan found a rising trend of CS in urban areas, and during 2012–13 the rate was about 25% [34]. Similarly, a study based on 80 Demographic and Health Surveys conducted in 26 countries in Southern Asia or Sub-Saharan Africa presented more simplified findings by accumulating CS in urban area with economic status where richest urban women were found as a more risky sub-group [35]. There could be number of reasons for increasing average annual change in the prevalence of CS among women who live in urban areas, who are better educated or from households with relatively high wealth status. Firstly, availability and accessibility of CS in urban areas may have an effect on its greater utilization. Secondly, generally women in urban areas are relatively better off, which can influence the affordability of CS. Thirdly, there are more private CS facilities in urban areas than in rural areas, and the burgeoning tendency of private facilities to use CS could have a major effect [11]. Fourthly, higher women employment rate in urban areas contribute to increased CS rate.

As expected, our results suggest a relatively high AARI and odds of CS among women who were overweight or obese. Our finding is consistent with the previous studies conducted in developed and developing countries [36–38]. The reasons for this increased rate of CS among overweight or obese women are not completely clear. However, previous studies indicate that this could be related to the increased complications in maternal soft tissue, fetal macrosomia in the intrapartum period, and majority of these deliveries occurred during the first stage of labor based on indications of dystocia and fetal distress [36, 39]. A wide range of adverse outcomes including excessive blood loss, pregnancy complication, gestational hypertension, gestational diabetes, preeclampsia, post-partum wound infection and endometrics among the overweight and obese maternal women were also reported in some studies [22, 37, 38, 40, 41]. Moreover, higher adverse neonatal outcomes including preterm birth, fetal distress, early neonatal death were found to be associated with maternal overweight and obesity during pregnancy [42]. Weight gain during pregnancy is another important factor. Previous studies suggest that obese women gain less weight during pregnancy than the normal requirement of 7–11.5 kg to optimize fetal growth leading to the further complication that warrant CS [43]. Besides, there is a common belief that every overweight or obese women must face adverse consequences during delivery and CS is the only potential remedy, though not required in all cases.

The reasons for rural to urban differences on the rate of CS use could be multifaceted and complex. There are socio-economic differences between rural to urban areas and so are in affordability of CS use. Also there is variation in availability/accessibility of CS facilities across rural and urban areas. The gross number of private hospital is generally higher in communities with more affluent people and they usually prefer private health facilities [18]. Cultural difference among both providers and patients may also impact on CS utilization. A portion of this variation could also be attributed to an increasing professional reliance on technology in urban areas. Also, reluctance among some healthcare providers to take any risks and a fear of litigation may contribute to increased utilization of CS. As apparent, most of these differences largely indicate effect of supply side factors compared to the ability to pay or women’s socio-economic status. While difference in CS use between urban and rural areas is quite easily understandable, the difference across rural areas warrants further investigation. Unequal distribution of trained midwives could be one of the reasons for variation among rural areas. Previous studies in Bangladesh [44] and other countries in South Asia [18] also found the regional and socio-economic disparities in CS use and availability of private and public hospital [18].

Our study showed that respondents aged ≤19 years and ≥35 years face a higher odds of using CS than those of 20–34 years. A recent meta-analysis [23] and studies conducted in developed and developing countries [44–46] also found similar result. Women in younger and advanced maternal age are more likely to suffer from obstetric and maternal complications than women in middle maternal age, and this may subsequently contribute to the increasing rate of CS use [46]. The exact age at which pregnancy complication may warrant CS is unclear. Moreover, there is lack of consensus about the healthy maternal age bracket. For instance, to define ‘advanced maternal age’ previous studies used a number of cut-off points such as 30 years [47], >35 years [48] and>40 years [23]. Apart from age, other factors such as overall health and well-being, chronic disease, ethnicity may impact on such a cut-off point.

We found a spatial variation in prevalence of CS use with relatively high levels of use among women in the Dhaka and Khulna regions. An earlier study conducted in Bangladesh using data of BDHS-2007 reported largely similar results [49]. We are, however, limited in ability to explain this finding, as, to our knowledge, there is very limited research on spatial variation. One likely explanation is that in Bangladesh retention of healthcare providers in some regions, often in remote areas, is a major problem. A significant number of posts are vacant in public health care facilities. Khulna and Dhaka divisions reported to have less vacant posts, resulting in the higher use of maternity care services in these administrative regions [50]. Also, relatively high number of functional emergency obstetric care facilities in public sector in Khulna region [51] and an overall higher concentration of CS services in capital city Dhaka–may also contribute to this variation.

CS is more prevalent among rich women where as poorer women reported relatively low rate. Our findings confirm the results of earlier studies conducted in Asia and Africa [35]. There are number of reasons that may explain such variation. The first is that people with higher socio-economic status generally choose private hospital where frequency of CS is more common. There may be enthusiasm among the well-off to adopt new medical interventions [52]. Secondly, poorer household fails to pay for the surgery and the extra cost associated with CS [52]. This may reflect a relatively low access to this life-saving procedure among the disadvantaged. Also a profit motive of health care providers and/or owners of private facilities could be an important factor. Additionally, rich are likely to be aware of their existing illnesses, and this knowledge may prompt them and doctors to consider CS. Sparse distribution of the necessary health facilities is another important reason [53].

Our findings suggest that having a formal education leads to a greater likelihood of CS use. This observation is consistent for Bangladesh [44, 49] and other developing countries [10, 24]. Education is intertwined with a range of other factors that lead such findings. One major factor is that formal higher education is a strong co-relate to women’s decision making autonomy [54]. Secondly, educated women may prefer CS because they believe it to be safer and interferes less with the workload and leisure time [10, 55].

A positive association between the use of antenatal care and delivery in CS has substantial public health implication. Antenatal visits are viewed as an essential measure for a complication-free normal pregnancy. It is not unlikely that these visits are used by the health care providers to motivate women to undergo CS. Indeed, some health care providers influence and prey on maternal behavior, such as fear of pain to promote CS [56]. Clearly, financial gain and/or excessive pressure of private employers are some likely reasons behind this malpractice.

Our study has several limitations. Firstly, besides the selective factors included in this analysis, health and gynecological factors are also significant predictors of women’s pregnancy delivery method. Secondly, the variation in jurisdictional accessibility, quality and cost of delivery services as well as the role of women in the decision-making process are likely to influence the delivery practices. Due to lack of relevant data, effects of these factors could not be examined. Our results might have been slightly affected by temporal elements arising from four waves of data collection at four different points of time. However, the multivariate model which we developed for examining significant factors of CS is free from this bias as we used only 2014 dataset for this model. CS in a nationwide study like ours could be affected by a range of factors, not all of those factors could be taken into consideration, as we used secondary data. Finally, the observed relationship between the outcome and covariates is co-relational only due to cross-sectional data structure.

## Conclusion

Around one-fourth of births were delivered by CS and this rate is on rapid rise. Maternal age, place of residence, socio-economic status, number of children delivered, number of antenatal visits and body mass index were significant predictors of CS. There were variations in CS use across geographical locations, population groups of varied socio-economic status. Women in the south-western region reported a higher AARI in CS than the women in other regions. These findings warrant further research to examine the precise reasons for excessive use of CS in some geographical locations, urban areas and by subset of women, and at the same time to assess if there is limited access for those who are relatively less advantaged even when CS is necessary. Rationalizing CS use is a complex task, which needs comprehensive research, policy and interventions. Women need to be made aware of the adverse effects of CS and the importance of controlling their body weight. In addition, service providers need to be better regulated to ensure that CS is only carried out when necessary and not for financial gains.
